# Conservation and tissue-specific transcription patterns of long noncoding RNAs

**DOI:** 10.3109/23324015.2015.1077591

**Published:** 2015-08-10

**Authors:** Melanie Ward, Callum McEwan, James D Mills, Michael Janitz

**Affiliations:** ^a^ School of Biotechnology and Biomolecular Sciences, University of New South Wales, Sydney, NSW 2052, Australia

**Keywords:** lncRNAs, comparative genomics, gene regulation, transcriptome, RNA-Seq

## Abstract

Over the past decade, the focus of molecular biology has shifted from being predominately DNA and protein-centric to having a greater appreciation of RNA. It is now accepted that the genome is pervasively transcribed in tissue- and cell-specific manner, to produce not only protein-coding RNAs, but also an array of noncoding RNAs (ncRNAs). Many of these ncRNAs have been found to interact with DNA, protein and other RNA molecules where they exert regulatory functions. Long ncRNAs (lncRNAs) are a subclass of ncRNAs that are particularly interesting due to their cell-specific and species-specific expression patterns and unique conservation patterns. Currently, individual lncRNAs have been classified functionally; however, for the vast majority the functional relevance is unknown. To better categorize lncRNAs, an understanding of their specific expression patterns and evolutionary constraints are needed.

## Introduction

Recent developments in RNA sequencing (RNA-Seq) technology have given scientists an in-depth view of the human transcriptome [1]. It is apparent that traditional views of RNA as merely an intermediary molecule between DNA and protein discredits the complexity of the human genome and ignores the pivotal role of noncoding RNA (ncRNA) as a regulatory molecule in essential life processes [2]. Despite merely a twofold increase in the number of protein-coding genes between the human genome and that of the common fruit fly, *Drosophila melanogaster*, these species exhibit dramatically differing levels of phenotypic complexity. To account for this disparity, there must exist a multi-level regulatory mechanism enabling such drastic diversity from a similar number of protein-coding genes.

**Figure 1.**
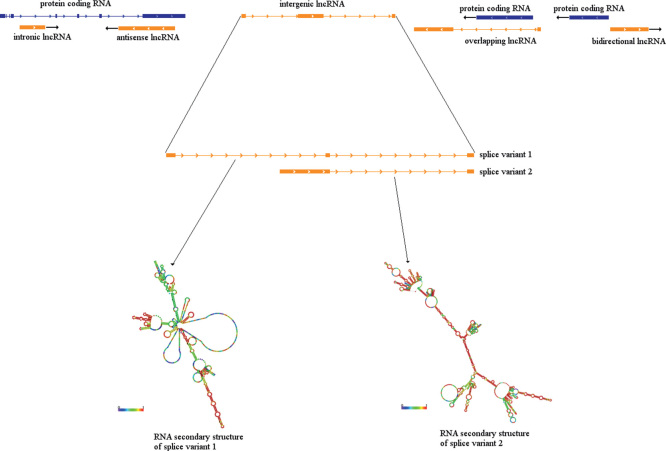
**Genomic localization-based classes of lncRNAs. Upper panel: *Intronic lncRNA:* the lncRNA is transcribed from an intronic region of a protein-coding gene. *Antisense lncRNA:* the lncRNA is transcribed from the strand opposite to protein-coding gene, with partial or complete overlap of any intronic or exonic regions. *Intergenic/intervening lncRNA*: the lncRNA is transcribed from a region located between other genes. There is no overlap with any protein-coding genes. *Overlapping lncRNA:* The intron of the lncRNA encompasses a protein-coding gene. *Bidirectional lncRNA:* The lncRNA shares its transcription start site with a protein-coding gene on the opposite strand. Arrows indicate orientation of transcription. Lower panel: lncRNAs can be alternatively spliced to produce numerous splice variants. Here, the intervening lncRNA is spliced to produce two variants. Each of these variants produces RNAs with unique secondary structure. The unique RNA secondary structure can determine function of the lncRNA isoform.**

There is a direct correlation between the proportion of ncRNAs in an organism’s genome and its developmental complexity [3]. The largest subclass of ncRNAs is long noncoding RNAs (lncRNAs). These are mRNA-like transcripts arbitrarily defined as being greater than 200 nucleotides long, with no protein-coding capacity, which however undergo alternative splicing and post-transcriptional processing [4]. Initially dismissed as ‘junk DNA’ where any transcription was interpreted an artifact of transcriptional noise, it has recently been shown that far more of the genome is pervasively transcribed than first hypothesized [5]. While they do not code for a protein, lncRNAs have been strongly associated with the regulation of epigenetic processes and expression of protein-coding genes. lncRNAs can be arranged as intergenic/intervening, antisense, intronic, overlapping and bidirectional, in relation to their localization to protein-coding genomic loci (Figure 1) [6]. There is now a growing wealth of data to suggest that lncRNAs possess biological function [7-9].

The dysregulation of lncRNAs expression has been implicated in a number of diseases across different tissue types. Merely 7% of disease-associated single nucleotide polymorphism (SNPs) is located within protein-coding regions compared to the 93% of SNPs that are found in noncoding regions [10]. Despite this asymmetry in SNPs distribution, the determination of lncRNAs role in disease pathogenesis remains difficult due to a lack of functional information prohibiting domain and functional prediction that is possible with protein-coding genes.

lncRNAs have been shown to be expressed in a distinct pattern across a number of tissue types. A number of lncRNAs have also been shown to be expressed in discrete cell types and within distinct subcellular structures [11,12]. These findings coincide with notions of lncRNA as regulators of gene expression in specific cell types. Thus, the identification and characterization of human lncRNAs with tissue-specific expression become essential in order to determine their relevant functions.

Another interesting property of lncRNAs is their rapid evolution across species. Previously, the conservation of sequence was thought to be evidence of functionality but lncRNAs have proved that this is not always the case. The tissue-specific expression patterns of lncRNAs, coupled with their distinctive conservation patterns, make lncRNAs a unique transcriptional element that warrants further investigation.

## Tissue-specific expression of lncRNAs

LncRNAs exhibit notably higher degree of tissue specificity when compared to protein-coding genes [13,14]. The apparent specificity of lncRNAs throughout various tissue and cell types has been repeatedly highlighted and is indicative of specific regulatory roles within essential cellular processes [11,15,16]. Indeed, if lncRNAs were merely the result of transcriptional noise we would expect little variation in expression levels between tissues [2]. A comparative study investigating tissue specificity of lncRNAs across 11 tissue types found that the majority of lncRNA expression was restricted to discrete tissue types with 67% of lncRNAs demonstrating a tissue-specific expression pattern and with 29% found to be expressed in only one discrete tissue type [17]. This widespread consistency of specific lncRNAs across different tissue types is suggestive of their specific biological function within the individual tissue. Despite this, little work has been done, as of now, in characterizing the expression profiles of tissue-specific lncRNAs beyond possible roles in disease pathogenesis, and in particular cancer [18].

### Brain

The brain is the most complex tissue in the human body. Beyond its billions of neurons, the brain comprises a number of other cell types, such as oligodendrocytes, astrocytes and microglia with heterogeneous distribution across anatomical subregions. Due to this complexity in terms of both structure and function, the brain requires a similarly complex regulatory system and as a result is the richest source of lncRNAs in the body [2]. LncRNAs play an essential role in the brain in terms of development, neuronal maintenance and function and have been linked to a number of neurodegenerative diseases [19]. When addressing the brain physiology of humans, the lncRNA repertoire is the greatest point of differentiation from other primates and other vertebrate species entirely due to the increased developmental complexity of the human brain [20]. Despite a high level of sequence similarity of protein-coding genes between humans and other primates we see far less conformity in the noncoding portion of the genome that is transcribed. Indeed the number of lncRNAs, particularly brain-specific, have been shown to directly increase in correlation with developmental complexity even as the number of protein-coding genes remains relatively unchanged [2].

There is a growing amount of data on the highly tissue-specific lncRNA transcripts located between protein-coding gene loci, known as long intervening ncRNAs (lincRNAs), and their role in regulation of fundamental cellular processes [14]. The lincRNAs are generally expressed at lower levels than protein-coding genes (∼10-fold lower) [14]; however, the brain transcriptome contains many unique lincRNA transcripts that are expressed at significantly higher levels than many protein-coding genes, such as the oligodendrocyte maturation-associated lincRNA (*OLMALINC*) [15]. *OLMALINC* is a primate-specific transcript that has been shown to play an essential role in the regulation of genes responsible for human oligodendrocyte maturation [15]. *OLMALINC* is highly expressed in the white matter of the human frontal cortex with expression levels of 71.5 fragments per kilobase of exon per million fragments mapped (fpkm) as determined by RNA-Seq. Such high level of expression indicates a strong regulatory role in oligodendrocytes, which comprise the majority of white matter. The differential expression of *OLMALINC* in gray and white matter (16.2 and 71.5 fpkm, respectively) demonstrates the dynamic nature of the brain transcriptome and the tissue specificity of lincRNAs.

A recent profiling of the transcriptome patterns of gray and white matter highlighted the tissue-specific nature of lincRNAs in a healthy brain [21]. The expression of lincRNAs differs significantly between gray and white matter and this is believed to be largely due to the nonconformity in cell populations between the tissues. Thus in each tissue type there exists divergent transcriptome profiles indicative of discrete roles in brain function for the different tissue types and provides evidence that lincRNAs function in a cell type-specific manner [21].

There is a growing need for the development of more comprehensive expression profiles of lncRNAs for all regions of the human brain [2]. Recent transcriptome analyses of the hippocampus and pre-frontal cortex of the adult mouse brain found highly specific lncRNA expression signatures within subregions of the brain and distinct neuronal populations [22]. A total of 2759 lncRNAs were found to be expressed in the hippocampus, 2561 in the pre-frontal cortex and of these 2390 lncRNAs were expressed in both regions while 24 were differentially regulated. The expression levels of the six highest differentially expressed lncRNAs were then analyzed in the cerebellum and striatum, and compared to that of the hippocampus and the pre-frontal cortex. The majority of these lncRNAs were found to be differentially regulated across all of the brain subregions. A further study using the Allen Brain Atlas showed lncRNAs to be expressed not only in specific subregions of the mouse brain but also in specific cell types and subcellular compartments [23]. The specific localization of lncRNAs supports the premise of their functionality.

The purported functional relationship between lncRNAs, as *cis*-regulatory elements, and adjacent protein-coding genes has also been observed in the human brain [9,24]. It has been found that many of these adjacent protein-coding genes have neurodevelopmental functions and the expression levels of adjacent lncRNAs consistently impact the transcription of the protein-coding genes. Despite this, no consistent pattern has emerged linking the transcription of lncRNAs and adjacent protein-coding genes [25]. These lncRNAs have also been indicated to have an integral role in the regulation of cellular differentiation of neuronal and glial cells, particularly during development [25].

Despite an incomplete annotation of the long noncoding transcriptome and a general lack of functional information, the dysregulation of tissue-specific lncRNAs has been strongly associated with a number of diseases [19]. The differential expression of lncRNAs in healthy and diseased states is shown through comparisons of the transcriptome profiles, which differ significantly in neurodegenerative diseases such as multiple system atrophy (MSA) [26] and Parkinson’s disease [27]. Despite consistent association between the dysregulation of brain-specific lncRNAs and neurological disorders [19,28], further research is required to individually categorize and ascertain the functions and molecular mechanisms of action of the dysregulated lncRNAs in order to determine their role in disease progression.

### Testis

Testis is a rich source of many unique lncRNA transcripts; however, very little is known about lncRNAs expressed solely in this organ. In-depth analyses of the testis transcriptome using RNA-Seq data have shown a widespread and diverse transcription of both protein-coding and ncRNAs [29]. The testis has two key functions: the secretion of sex hormones and spermatogenesis. The production of spermatozoa is a complex biological process involving multiple stages controlled by epigenetic and molecular mechanisms at both transcriptional and post-transcriptional levels [30]. The need for such regulation has been suggested as a reason for the diversity of the testis transcriptome with specific lncRNAs predicted to play key regulatory roles [14].

A comparative study investigating the five most common cell types involved in spermatogenesis found that in addition to expressing a greater palette of lncRNAs transcripts than cells of the brain or liver, the expression of unique lncRNAs differed significantly between the cells of the testis producing highly specific expression patterns [29]. This was particularly pronounced in spermatids and spermatocytes, which exhibited the highest levels of lncRNA transcription [29].

Currently there are limited studies into human testis-specific lncRNAs expression and as a result we must rely on animal models. A recent study produced lncRNA expression profiles for the testis of a neo-natal and adult mouse [31]. This study identified over 3000 differentially expressed lncRNAs between the neo-natal and adult mice [31]. These dramatic differences in lncRNA expression could indicate a significant biological role for lncRNA during the testis post-natal development. Furthermore, lncRNAs were found to exhibit a greater spatial and temporal specificity than protein-coding genes consistent with previous studies and supportive of a cell type-specific regulatory role.

### Liver

The role and function of lncRNAs in the liver is largely unknown but the dysregulation of specific transcripts has been associated with liver diseases such as hepatocellular carcinoma [32] and nonalcoholic steatohepatitis [33]. Liver-specific lncRNAs have also been implicated in the regulation of processes such as lipid metabolism. Liver-specific triglyceride regulator (*lncLSTR*) was found to regulate the clearance of triglyceride and help maintain systemic lipid homeostasis through a novel lncRNA signaling pathway. Its apparently key role in this crucial metabolic process highlights the potential physiological importance of lncRNAs in the liver.

### Heart

Little is known about the role of lncRNAs in the heart; however, a heart-specific lncRNAs has been found to be involved in cardiac development. FOXF1 adjacent noncoding developmental regulatory RNA (*Fendrr*) is a lateral mesoderm-specific lncRNA that is essential for the development of the heart wall in mouse and was shown to have an orthologous transcript in humans [34]. *Fendrr* was found to modulate chromatin signatures that define gene activity by binding directly to the histone-modifying complexes Polycomb repressive complex 2 (PRC2) and histone–lysine N-methyltransferase 2A (KMT2A), which play a central role in the activation of genes responsible for cell differentiation and lineage commitment. PRC2 and KMT2A act as a repressor and activator of cellular proliferation, respectively, in the heart during embryonic development. The knockdown of *Fendrr* in mice was shown to be lethal to the embryos due to heart wall deficits and significantly impaired heart function demonstrating its importance for normal heart function.

### Skeletal muscle

Long intergenic ncRNA, muscle differentiation 1 (*Linc-MD1*) has been identified to have a significant role in myogenesis through its control of muscle differentiation [35]. *Linc-MD1* expression is temporally dynamic in order to control the progression through the stages of muscle differentiation where it functions as a competing endogenous RNA for the binding of the microRNAs (miRNAs) *miR-133* and *miR-135*. The two miRNAs regulate the binding of the transcription factors that promote muscle differentiation. Hence, *Linc-MD1* plays a crucial role in the regulation of muscle terminal differentiation through its action as part of a network of regulatory interactions.

### Retina

Several retina-specific lncRNAs in mice have been identified and determined to be of functional importance in retinal cell development and differentiation. Six3 opposite strand transcript (*Six3OS*) is promoter-associated lncRNA found to play a role in the regulation of retinal cell differentiation through knockdown and overexpression studies [36]. *Six3OS* was also shown to modulate the expression of associated protein-coding genes through the recruitment of histone modification enzymes. *Six3OS* acts as a molecular scaffold that leads to the recruitment of histone modification enzymes. A retina-specific lncRNA ventral anterior homeobox 2, opposite strand (*Vax2os*) was also shown to regulate the cell cycle during mammalian retina development [37]. Overexpression of this transcript during the early stages of development was associated with a reduced rate of retinal cell proliferation. *Vax2os* is so far the only example of a cell type-specific lncRNA regulating the cell cycle during mammalian development.

## Rapid evolution of lncRNAs

LncRNAs show very little conservation in their sequence and they evolve rapidly [38-40]. The predicted amount of shared functional sequence decreases dramatically as the divergence between mammalian species increases, suggesting a very high rate of sequence turnover [41]. The rate of nucleotide substitution in protein-coding sequences is ∼ 10%, whereas noncoding sequences have a substitution rate of 90%.

The rapid evolution of lncRNAs originally led to the assumption that they were nonfunctional. Nonfunctional sequences tend to display a similar rate of sequence change when compared to evolutionarily neutral sequences [42]. However, lncRNAs have demonstrated more constraint than random intergenic regions [43]. Ancient lncRNAs (minimum of 90 Myr) show higher levels of long-term exonic sequence conservation than untranslated regions, with the oldest presenting similar levels of constraint with protein-coding exons. In comparison, young lincRNAs (under 25 Myr) show lower levels of exonic sequence conservation than random intergenic regions [39]. This may be due to the fact that young genes demonstrate rapid evolution [44]. Young genes are more susceptible to variable selection pressures than well-established genes [45]. Interestingly, lncRNAs with multiple exons appear to demonstrate greater evolutionary constraints within exons [46].

## Conservation beyond the primary sequence

The sequence of RNAs can differ whilst their secondary structure can be conserved [47,48]. Many lncRNAs showed a number of correlated positions that could be the result of conserved secondary structures (Derrien *et al.* 2012). One of the well-characterized lncRNAs, HOX transcript antisense RNA (*HOTAIR*), is believed to have conserved structures but divergent sequences across species [44]. RNAs can form a variety of structures such as tetraloops [49], GU base pair motifs [50], adenosine platforms, helixes and tandem repeats [51]. These motifs have demonstrated sequence conservation, for example the hairpin loop and the tRNA-like structure in the lncRNA metastasis-associated lung adenocarcinoma transcript 1 (*Malat1*) [52]. The majority of the helixes appear to be conserved across a variety of species, in comparison to the base paired regions, which are not so well conserved [53]. This theory is supported by the fact that many lncRNAs with differing sequences are able to bind to the same protein [54,55].

The functional role of the lncRNA may also be conserved. One established lncRNA is X-inactive-specific transcript (*Xist*), which is involved in X-chromosome inactivation. The function of *Xist* is conserved across mammals, even though the sequence is evolving at a high rate [56]. In addition mouse and zebra fish lncRNAs, involved in embryonic development, did not have conserved sequences, whereas the function appears to be conserved [57]. If the functional roles of lncRNAs are conserved across species, then it is most likely that their loci will also be conserved [38]. Indeed, studies have found that lncRNAs have conserved synteny across a range of species [39,58].

## LncRNA evolution in primates

King and Wilson first proposed that the major biological differences between humans and chimpanzees are due to gene regulation, not differences in sequence [59]. There are probably too few changes in the amino acid sequence of proteins to result in the phenotypical differences between humans and chimpanzees [20]. In fact, a larger number of protein-coding genes are conserved for primates when compared to lncRNAs; 92% of human intergenic lncRNAs are expressed in chimpanzee or bonobo and ∼ 72% are expressed in the macaque. In comparison > 98% of protein-coding genes is conserved for all primates [39].

It is believed that human brain evolution has occurred through changes in noncoding parts of the genome [60]. The human brain is in fact a rich source of lincRNAs, further supporting this theory [21,26]. The majority of gene expression differences between the brains of humans and nonhuman primates involved upregulation of gene expression in humans [61]. While this may be due to higher levels of neuronal activity, it has been found that genes critical for neural development are upregulated across mammals [62].

Brain growth patterns vary across primate species [63] and humans show a unique pattern of expression [61]. The expression pattern of genes in the chimpanzee brain cortex is more similar to gene expression patterns in macaques than humans [64]. This indicates an increase in the rate of evolution in gene regulation in the human lineage [64]. The expression of human-specific genes was greater in the frontal lobe in comparison to the hippocampus and caudate. This suggests that the majority of evolutionary change in the human brain was focused in the frontal lobe [20]. Genes in the frontal lobe that are associated with neuron projections, neurotransmitter transport, synapses, axons and dendrites, as well as genes implicated in schizophrenia showed increased connectivity in the human brain when compared to chimpanzees and macaques [20].

One example of a noncoding gene that is thought to have evolved a unique function in humans is the human accelerated region 1 (*HAR1*). It has been suggested that *HAR1* has been evolutionarily selected for increased stability [65]. It is believed that A/T to C/G substitutions led to a more stable secondary structure in *HAR1* [62]. Forkhead box protein P2 (*FOXP2*) and abnormal spindle-like microcephaly associated protein (*ASPM*), which are involved in speech production and brain size respectively, have undergone the same kind of evolutionary change [66,67].

## Methods of detecting lncRNAs

RNA-Seq is a high-throughput next-generation sequencing technique that is capable of measuring RNA expression levels and providing an accurate picture of the transcriptome [68]. RNA-Seq has numerous advantages over other transcriptome profiling techniques such as microarrays. RNA-Seq has a higher resolution, lower levels of background noise, lower requirement of input RNA and can detect a greater range of expression levels [69]. The most important aspect of RNA-Seq is that it can be used to assemble transcriptomes *de novo*; this allows for the discovery of un-annotated transcripts and novel splicing events [69]. This ability makes RNA-Seq an ideal tool for the identification species- and tissue-specific lncRNAs, many of which have not been previously annotated.

More recently, slight modifications of the template preparation stage of RNA-Seq have allowed for the strand of origin from which an RNA molecule is transcribed from to be tracked, thus allowing for the identification of antisense transcription. These techniques are known as strand-specific RNA-Seq [70,71]. While a multitude of different strand-specific RNA-Seq exist, currently the most widely used is the dUTP second-strand marking method [70]. Strand-specific RNA-Seq techniques allow for the identification of antisense transcripts and this feature is particularly relevant to lncRNAs. Examples of antisense lncRNAs include TSIX transcript, XIST antisense RNA (*TSIX*) [72] and the beta-site APP-cleaving enzyme 1 antisense RNA (*BACE1-AS*) [73]. It is estimated that between 20–30% of human transcripts have an antisense partner [74,75]. Further, the amount of antisense transcription will vary from cell type to cell type [76]. Another important technical advance concerning RNA-Seq is the use of ribosomal depletion to select the RNA fraction for sequencing rather than selecting only those transcripts that are polyadenylated. Ribosomal depletion removes ribosomal RNA from the samples, allowing for the selection of both polyadenylated positive (poly(A)+) and polyadenylated negative (poly(A)−) fractions for sequencing [77]. This is important as large amounts of transcriptional output in eukaryotic cells is poly(A)− [78]. As ribosomal depleted strand-specific RNA-Seq becomes the standard for all transcriptome-profiling experiments, it is expected that more lncRNAs will be found throughout different tissue types in the human body.

Raw RNA-Seq data needs to be processed and analyzed to answer all sorts of bioinformatics enquires, including investigation of gene/transcript expression levels, detection of alternative splicing events and identification of unannotated genes/transcripts. In brief to analyze RNA-Seq data, first the reads must be mapped to the reference genome, next transcripts are assembled followed by a differential expression analysis. A common workflow currently used by researchers is known as the Tuxedo suite, which utilizes the software packages Tophat, Cufflinks and Cuffdiff [79]. This workflow is ideal for the identification of lncRNAs as it has the ability to identify novel splicing events and un-annotated transcripts down to the resolution of a single base. It also takes advantage of data generated by ribosomal strand-specific RNA-Seq to locate antisense transcripts.

A typical RNA-Seq experiment will produce vast amounts of data. Generally it is not feasible to analyze data on a personal computer due to limitations in storage size and raw processing power. These problems can be overcome through the use of high-performance computing (HPC) clusters. A HPC cluster consists of multiple nodes, with each node containing one or more central processing units (CPUs), each with numerous cores. HPC clusters are normally a resource shared across a major institute such as a university or hospital. Another alternative is to take advantage of cloud computing services such as Amazon Web Services (AWS) (http://aws.amazon.com/). AWS allows researchers to dynamically adjust the computing power and storage requirements based on current requirements and has potential computing power much larger than any HPC cluster.

## Concluding remarks and future directions

Only recently has technology been able to identify lncRNAs using high-throughput methods such as RNA-seq. Questions still remain as to how many of the proposed lncRNAs are functional, what that function is and the role that they have played in evolution. More knockdown and overexpression studies are necessary to explore the diverse roles that lncRNAs possess. For example, the overexpression of the 3’UTR region of the phosphatase and tensin homolog pseudogene 1 (*PTENP1*), through retroviral vectors, revealed its role in the regulation of the phosphatase and tensin homolog (*PTEN*) [80]. RNAi of *OLMALINC* in human oligodendrocytes [15] revealed the perturbation of the expression of genes involved in the maturation and myelination of oligodendrocytes. A systematic approach is needed to attempt to elucidate the function of various lncRNAs, which could prove difficult due to the species and tissue specificity of many lncRNAs.

In order to better determine lncRNA role as part of a regulatory network it is essential to produce comprehensive, functional annotations for lncRNAs similar to those that exist for protein-coding genes. This is especially relevant for those novel lncRNAs associated with human diseases. As a result of advances into ncRNA research, there are several public databases of annotated lncRNAs; however complete functional characterization of all lncRNAs is needed beyond merely basic sequence and transcript information [81]. A number of lncRNA databases currently exist, each with different focuses which determines their utility. This includes LNCipedia with broad coverage of a high number of lncRNAs, lncRNA database (lncRNAdb) providing in-depth annotation of a variety of different lncRNAs and GermlncRNA with a tissue-specific catalogue of lncRNAs.

The lncRNAdb (http://www.lncrnadb.org/) provides a summary of known eukaryotic lncRNAs. lncRNAdb differs from many other databases as entries must be supported by literature and they do not pull their data from unconfirmed sources to ensure validity [82]. Thus, lncRNAdb serves as a reliable resource for exploration of eukaryotic lncRNAs; however it represents only a small fraction of currently annotated lncRNAs. The database currently contains 287 eukaryotic lncRNAs that have been manually curated and described independent of scientific literature [83]. It provides information on lncRNA function, sequences, expression data and relevant supportive literature. Of these, 100 lncRNAs have had function determined through direct *in vitro* and/or *in vivo* experiments.

GermlncRNA (http://germlncrna.cbiit.cuhk.edu.hk/) is a web-based lncRNA catalog containing annotations of male germ-cell specific lncRNAs [84]. This catalog currently contains 110476 annotated lncRNAs and 2790 novel lncRNAs, the latter classified as novel as they were unannotated in any of the public genomic databases. The database was created through the integration of male germ transcriptome profiles from microarray, RNA-Seq and GermSAGE studies [84]. A tissue-specific focus allows for more comprehensive gene coverage, especially important for the testes, which are a rich source of lncRNAs.

LNCipedia (http://www.lncipedia.org/) is a comprehensive database for annotated human lncRNAs generated through the incorporation of data obtained from a number of different sources. This allowed for a rapid increase of the gene entries from 21488 annotated lncRNAs in LNCipedia v.1.0 to 111685 annotated lncRNAs in LNCipedia v.3.1. [85]. Along with sequence/transcript information, secondary structure and protein-coding potential are explored in detail for many of the cataloged lncRNAs [86]. A strategy to detect lncRNAs with protein-coding potential has been integrated within the database, which reanalyzes the mass spectrometry data publicly available from the PRIDE database. The wide scale of LNCipedia allows for incorporation of its content into large genomic projects, including development of customized microarrays allowing genome-wide surveys of lncRNA expression.

These databases were created through the integration of pre-existing public resources. While this allows for large amounts of information to be shared and combined, it also led to lncRNA predictions that greatly vary between individual repositories. This is due to differences in methodology, classification and assembly algorithms, which result in many lncRNAs to be missed or improperly categorized [81]. Constant verification is required to ensure the validity of the database, which is particularly difficult to achieve in large lncRNA databases. This remains an issue with the number of lncRNAs being annotated constantly increasing but experimental functional characterization lagging behind. Before function can be determined for all annotated lncRNAs, for example utilizing knockdown and overexpression approaches, a complete and comprehensive catalog of evolutionary conservation and tissue-specific expression for these transcripts must firstly be produced.
